# Localization of external urethral orifice in coronary sulcus during urethroplasty in case of severe hypospadias accompanied by prostatic utricle cyst

**DOI:** 10.1186/s12894-021-00913-5

**Published:** 2021-11-05

**Authors:** Jun Lu, Junjie Cen, Wenwei Wang, Hongwei Zhao, Pengju Li, Jiacong Mo, Zhenhua Chen, Yiming Tang, Jinhuan Wei, Junhang Luo, Shiying Huang, Yong Fang

**Affiliations:** 1 Department of Urology, First Affiliated Hospital of Sun Yat-Sen University, Guangzhou, 510080 People’s Republic of China; 2 Department of Urology, Affiliated Yantai Yuhuangding Hospital, Qingdao University, Yantai, 264000 Shandong People’s Republic of China; 3 School of Traditional Chinese Medicine, Southern Medical University, Guangzhou, 510515 People’s Republic of China

**Keywords:** Hypospadias, Prostatic utricle cyst, Urethroplasty, Epididymitis

## Abstract

**Background:**

To explore whether opening the external urethral orifice in the coronal sulcus can reduce the incidence of epididymitis after operating on hypospadias with prostatic utricle cyst (PUC) connecting to the vas deferens. Group A consisted of 3 patients with severe hypospadias and PUC undergoing cystostomy, hypospadias correction and urethroplasty, along with the relocation of the external orifice of the urethra to the coronal sulcus. Group B consisted of 4 patients having initial hypospadias repaired with meatus in the orthotopic position in the glans, presenting with multiple epididymitis after hypospadias surgery and unsuccessful conservative treatment. MR confirmed that all the Group B patients had PUC connecting to the vas deferens. Group B patients underwent urethral dilatation along with urethral catheterization, cutting of the original corpus cavernosum that encapsulated the urethra, and extension of the position of the external urethral orifice to the coronal sulcus.

**Results:**

In group A, 3 children underwent bladder fistula removal 2 weeks after the operation. The penis developed normally without any complications. Four children in group B underwent stent removal 12 weeks after operation, and one patient was still stenosed and dilated again. All patients in group B were followed without epididymitis recurrence.

**Conclusions:**

For patients with hypospadias complicating with a PUC, connecting to one side of the vas deferens, the positioning of the external urethral orifice in the coronary sulcus would be helpful to reduce the occurrence of epididymitis.

## Introduction

The prostatic utricle cyst (PUC) is a cystic structure located in the anterior rectal bladder and posterior spermatorrhea, which originates from incomplete degeneration of the Mullerian duct. The incidence of simple PUC in children is low, but in children with hypospadias and sex organ abnormalities, the occurrence increases to 11–14%, while in children with severe hypospadias, it can be as high as 57% [1–4]. However, due to the lack of systematic examination before hypospadias surgery, the incidence of hypospadias accompanied by PUC is often underestimated [5]. The majority of patients are asymptomatic, but the PUC may be associated with recurrent urinary tract infections, pain, abdominal mass, stone formation, pseudo-incontinence and epididymitis [6, 7]. Especially for patients with PUC and hypospadias, urethroplasty can increase the incidence of epididymitis. The specific reasons are as follows: due to the location of the vas deferens, two types of PUCs can form: one where the vas deferens opens to the PUC and another where the vas deferens is not connected to the PUC. For the first type, urinary pressure increases after urethroplasty, and urine can easily flow back into the vas deferens, leading to epididymitis [4]. In addition to the length of the urinary pathway requiring urethroplasty, another important factor to consider when performing hypospadias surgery is the location of the external urethral orifice. At present, there is an increasing demand for hypospadias surgery, making it necessary to perform more research on this surgery.

## Materials and methods

**Fig. 1:**
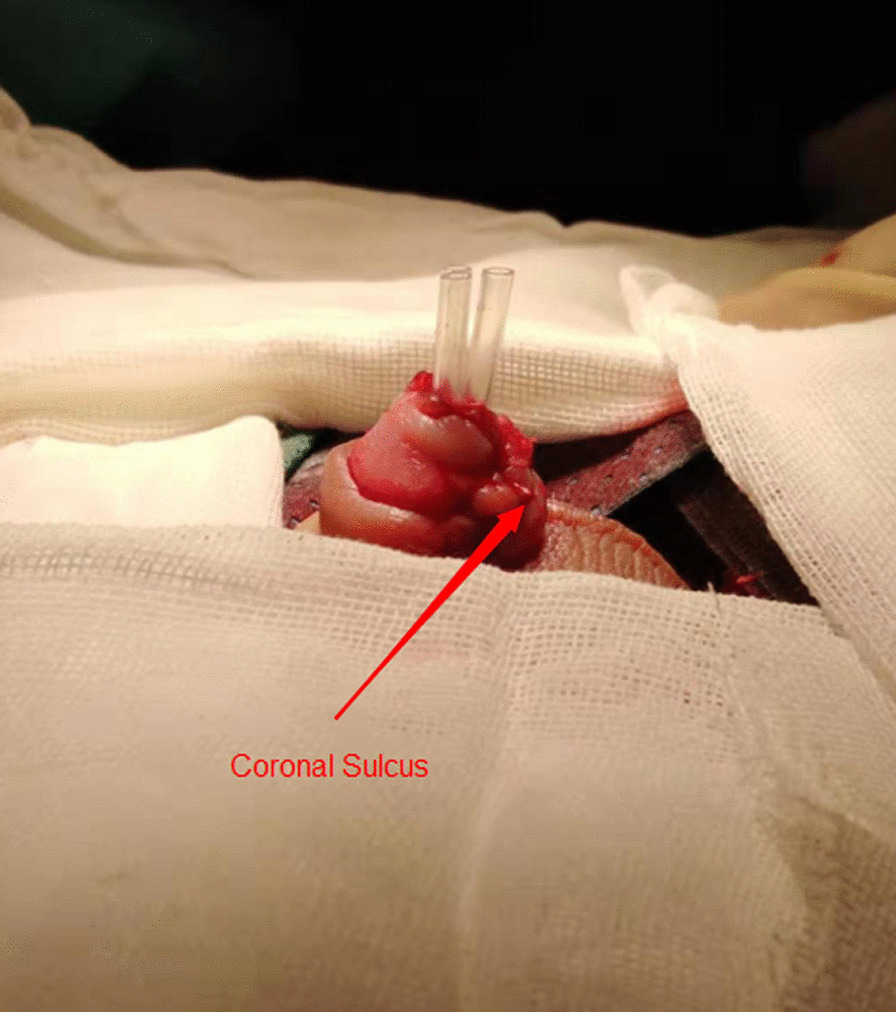
In patients with hypospadias without PUC, the external orifice of the urethra selected for urethroplasty is located at the front of the glans, surrounded by the glans cavernous body

**Fig. 2:**
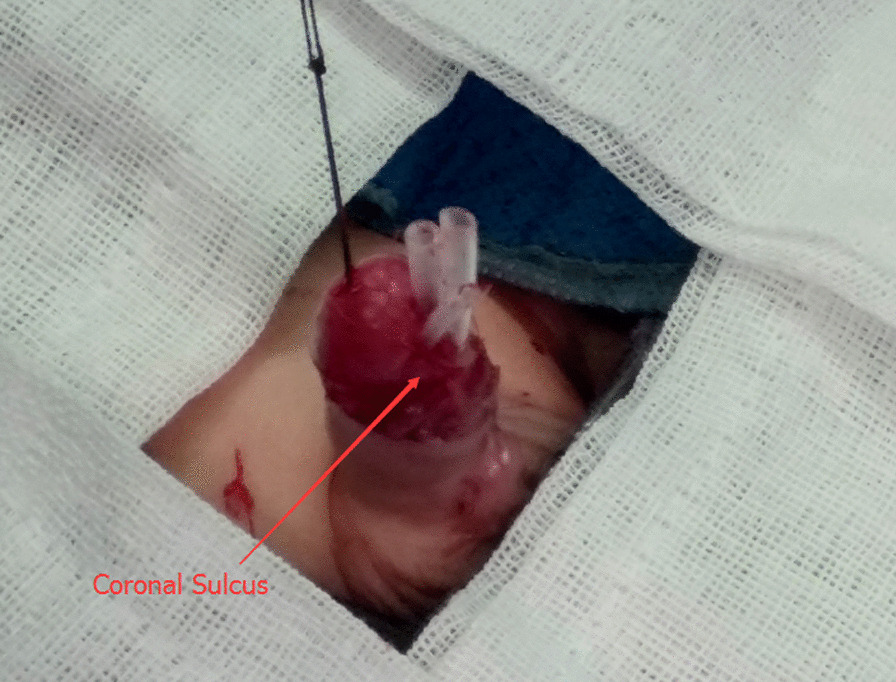
In patients with severe hypospadias complicated with PUC, the urethral orifice chosen is close to the coronal sulcus without enclosure by the glans cavernosum during urethroplasty

The MAGPI (meatal advancement and glanuloplasty incorporated) is an urethroplasty technique, and its key steps include moving the urethra opening to the normal position of the penile glans, surrounded by the glans cavernosum (Fig. 1). However, this method can cause increased pressure on the urethra, which can increase the possibility of developing epididymitis in patients with severe hypospadias and PUC (Group A in our study). Therefore, for these patients,our hospital chose to position the urethral opening, not in the median position of penile glans through the glans cavernosum, but near the coronal sulcus, without enclosure of the glans cavernosum. This is similar to Denis-Browne surgery (Fig. 2), which helps to alleviate the relative urethral pressure, thus reducing the occurrence of epididymitis. For the patients with epididymitis after urethroplasty, whose PUCs connecting to the vas deferens, we dilated the external urethral orifice to the adjacent coronal sulcus and tear the corpus cavernosum, as the corpus cavernosum was made to enclose the urethra in previous operations. Through this method, we were able to achieve successful clinical results. The following report details our methods and results.

**Fig. 3:**
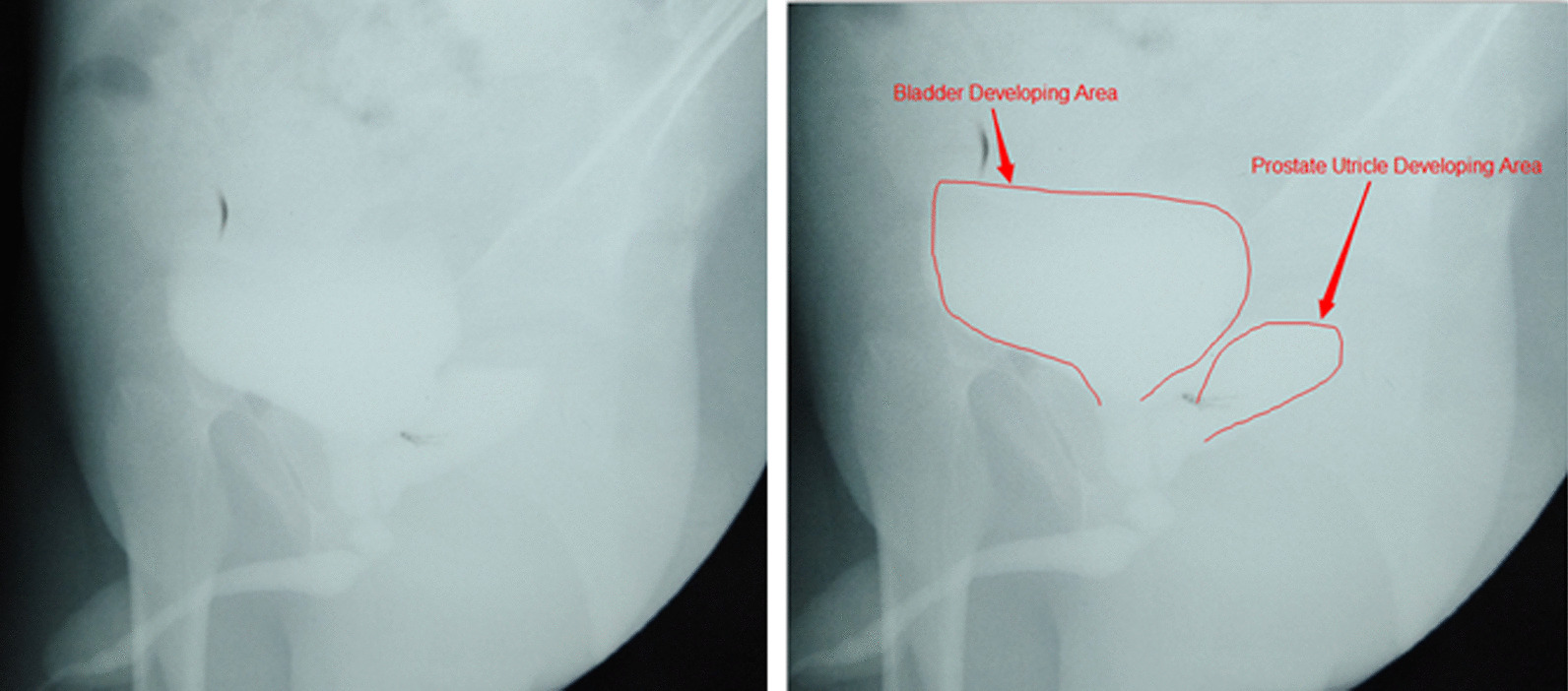
In group B, the original and enhanced portrait urethrogram of the patients with severe hypospadias and PUC showed1.1 cm × 2 cm × 3.5 cm, PUC posterior to the bladder

**Fig. 4:**
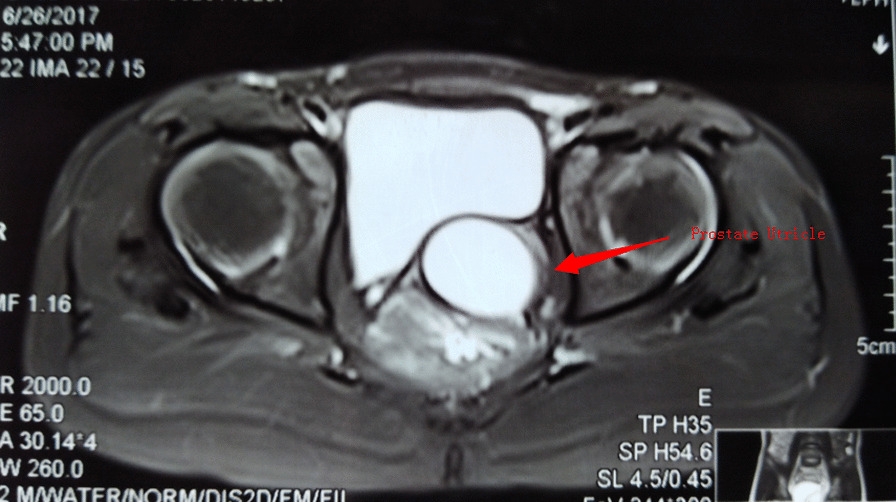
In group B, magnetic resonance imaging of severe hypospadias with PUC revealed cystic lesions in the left posterior bladder, about 2.6 cm × 1.2 cm × 3.8 cm in size

Three cases of hypospadias complicated by the presence of a PUC were operated on for the first time in the group A (Table 1), which accounted for 9.4% of the total corresponding time’s cases (32cases). Hospitalization occurred during 2011–2012 and the patients ranged in age from 3.1 to 3.6 years, with an average age of 3.3 years. They all displayed severe hypospadias, with 2 cases of penile-scrotal hypospadias and 1 case of perineal hypospadias. Renal Bladder Ultrasound and MRI showed that the PUC was round or oblong, with an average size of about 2.3 cm × 2.7 cm × 3.2 cm, and one side of vas deferens was connected with PUC in all 3 patients. Four patients (group B) with multiple epididymitis after hypospadias surgery and unsuccessful conservative treatment were admitted to the hospital from 2006 to 2017 (Table 2), which accounted for 44.4% of the total corresponding time’s cases (9 epididymitis after hypospadias cases). The age varied from from 3.5 to 8.6 years, with an average age of 6.9 years. There was no routine examination before hypospadias surgery for these patients with PUC, 1 case of penile hypospadias, 1 case of penile-scrotal hypospadias and 2 cases of perineal hypospadias. Renal Bladder Ultrasound, voiding cystourethrography (VCUG) (Fig. 3) and MRI (Fig. 4) were performed in group B, and MRI showed that the average size of PUC was 2.6 cm × 2.9 cm × 3.5 cm, and one side of vas deferens opened to the PUC in all 4 patients. The study was approved by the ethics committee of the First Affiliated Hospital of Sun Yat-sen University. The informed consent was obtained from each patient’s parents. This study adhered to the guidelines of Chinese diagnosis and treatment of urological diseases. In group A, after filling the bladder with water, a suprapubic tube and drainage were performed 2 cm above the pubic symphysis; the skin was cut under the coronal sulcus, subcutaneous tissue was dissected and subcutaneously separated outside the albuginea. Fibrous bands restricting penile extension were completely separated and released to make the penis straight. After correcting the lower curvature, the average length of the defect urethra was about 5.6 cm in these three cases. Then urethroplasty was performed on the urethral plate and the prepuce was grafted, and the average width of the urethral plate was about 1 cm. The thin prepuce inner plate was transferred to the ventral side of the penis and wrapped around 3–4 stents with side holes. The graft prepuce margin was sutured continuously with 6–0 absorbable sutures. The proximal end was sutured intermittently with the original urethral orifice by varus suture. The distal end was reconstructed with a new urethral external orifice by separating the inferior dermal bridge of the glans, which was located in the coronal sulcus, without encapsulation of the glans corpus cavernosum, which is different from the traditional MAGPI. Penile subcutaneous tissue was sutured. The mesh yarn wrapped around the penis and was fixed with gauze strips.

**Table 1:** The individual patient clinic data of group A

Group A	Age (years)	Size of puc (cm)	TH	UOLO	EOO	LFU (years)
Patient 1	3.1	2.5 × 2.3 × 3.0	Penile-scrotal	Coronal sulcus	No	6.5
Patient 2	3.2	2.1 × 2.8 × 3.5	Perineal	Coronal sulcus	No	5
Patient 3	3.6	2.3 × 2.3 × 3.1	Penile-scrotal	Coronal sulcus	No	7

TH: the type of hypospadias; UOLO: urethral orifice location in operation; EOO: epididymitis onset or no; LFU: the length of follow up

**Table 2:** The individual patient clinic data of group B

Group B	Age (years)	Size of puc (cm)	TH	UOLOO	TEAOO	AIHS (years)	TBEX (years)	LFU (years)
Patient 1	3.5	2.6 × 1.2 × 3.8	Perineal	Top of glan	3	2.0	2.7	5.5
Patient 2	7.3	3.3 × 4.1 × 4.2	Perineal	Top of glan	5	3.2	3.8	2
Patient 3	8.6	1.1 × 2 × 3.5	Penile-scrotal	Top of glan	6	3.5	4.2	10.5
Patient 4	8.2	3.4 × 4.2 × 4.1	Penile	Top of glan	5	2.7	3.5	12

TH: the type of hypospadias; UOLOO: urethral orifice location in original operation; TEAOO: times of epididymitis after original operation; AIHS: the ages at the initial hypospadias surgery; TBEX: times of beginning to experience epididymitis; LFU: the length of follow up

**Fig. 5:**
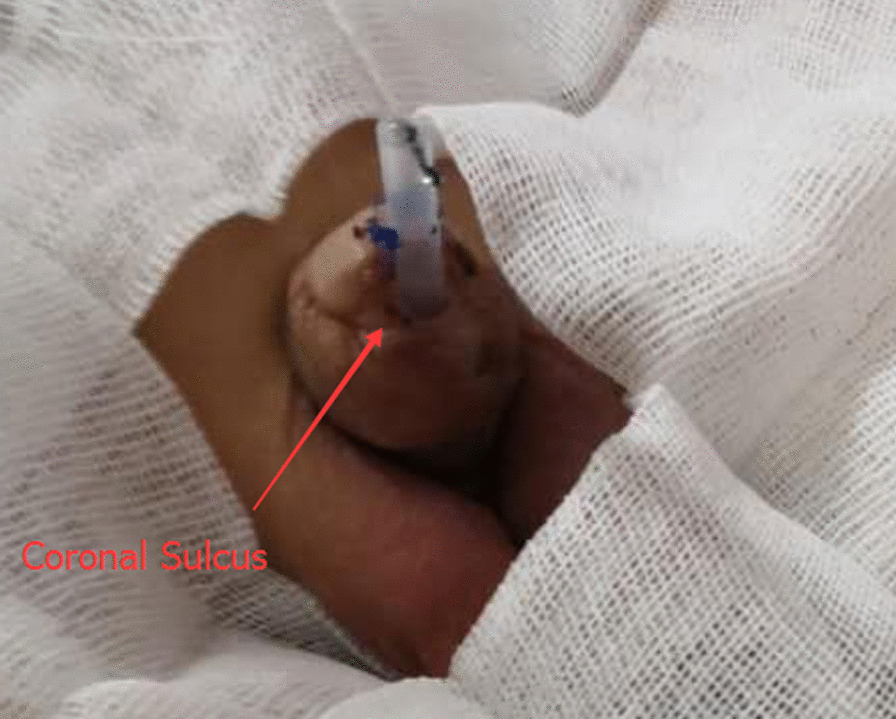
The original glans, cavernosum was torn to the coronal sulcus and a 5 cm long silica gel stent tube was fixed to the glans

In group B, the original urethral orifice was located at the top of the glans. When the urethral dilatation probe was inserted, stricture of the external urethral orifice was found in all cases. First, the filamentous probe was inserted through the urethra, and then F8-F18 urinary dilatation strips were inserted sequentially. The ventral margin of the urethral orifice was pulled to the ventral side of the glans, and the original glans cavernosum was torn to the coronal sulcus. In this way, the external orifice of the urethra was no longer surrounded by the glans cavernosum, and there was a gap of about 0.5 cm. After the dilatation, a 5 cm long silica gel stent tube was fixed to the glans (making the urethra about F16 in diameter) (Fig. 5).

## Results

The suprapubic tube was removed 2 weeks after the initial operation in group A, and the internal stent was removed 4 weeks after the operation. The external genitalia developed normally and there were no complications during the follow-up period of 5–7 years. Four children in group B were treated, with stent removal 12 weeks after the operation, and one patient was stenosed and dilated again. All patients in group B were followed up for 2–12 years without epididymitis relapse, and penile erection as well as ejaculation were normal later in adulthood.

## Discussion

Normally, Mullerian inhibiting factor (MIF) prevents the development of the female reproductive system in embryos around the 10th week of development. When the secretion of MIF is insufficient or the urogenital sinus is not fully developed in a way that allows for the formation of male reproductive organs, the Mullerian tube can degenerate incompletely and form a cystic dilatation connected to the prostatic urethra, forming PUC or cyst. Cysts are small and asymptomatic and are often found in physical examination. They can also be secondary to infection, stones or obstruction. Additionally, urinary tract infections, epididymitis and scrotal abscesses can occur repeatedly [8], and there are even reports of malignant transformation of prostate cysts [9]. About 90% of PUC cases also display hypospadias or ambiguous external genitalia [10]. The end of the vas deferens (ejaculatory duct), normally opening in the verumontanum, can open into the cyst. According to Monfort [11], in 4 out of 5 cases, the vas deferens entered the cyst. Ritchey et al. [12] explained that the verumontanum originating from the urogenital sinus was affected due to the abnormality of the urogenital sinus and the formation of PUC, where the ejaculatory duct and vas deferens implant ectopically. Postoperative treatment of hypospadias is often complicated by urethral stricture up to 7–12% [13–15]. With the increase of urethral pressure after urethroplasty, epididymitis can often occur after undergoing surgery to remedy hypospadias with a PUC.

PUC is often found in the course of hypospadias surgery when it is difficult to insert a catheter, or when complications such as epididymitis and pelvic inflammatory occur after urethroplasty. Few PUCs are confirmed before urethroplasty. However, if the size and location of cysts are not known before an operation, it can cause difficulties during the operation and affect the success of the operation. Therefore, it is necessary to determine whether there is a PUC before the operation for hypospadias, especially for severe hypospadias. Shima et al. [16] reported that PUCs occurred in 13.9% (21/151) of penile-scrotal and perineal hypospadias patients. Therefore, the incidence of severe hypospadias, especially perineal hypospadias with PUC is high, and relevant imaging examinations should be performed before operating.

For the development of normal penis shape after the operation, the prevalent hypospadias surgery generally places the external urethral orifice in the front of the glans, surrounded by the glans cavernous body. The advantage of this external urethral orifice is that there is a cavernous body in the urethral orifice. The pressure of the cavernous body contraction is conducive to straightening the urethra and making the urine travel distance longer. However, the disadvantage is that it increases the pressure on the urethra during micturition, which is related to the poor compliance of the external urethral orifice in the glans. Compliance means the tendency of an organ to resist deformation by a force [17]; obviously, encapsulation of the external orifice of the urethra by the glans cavernosum reduces urethral compliance. Furthermore, the new external urethral orifice protruded from the glans tends to form scar stricture or even atresia, and the urethral orifice stricture accounts for about 50% of the total urethral stricture after hypospadias surgery [18], Lichen sclerosus may be an important factor in distal urethral stricture [19]. This stricture can also cause increased urethral pressure, therefore, urine can more easily flow back to the testis, causing epididymitis attacks, especially in patients with hypospadias complicated with PUC. For these reasons, we advocate that the external urethral orifice should be placed in the coronal sulcus in these patients. When this method was tried in our hospital, for the 3 patients in group A, the external orifice of the urethra was placed near the coronal sulcus instead of passing through the glans cavernosum in their initial operation, and no epididymitis occurred. The 4 patients in group B suffered from recurrent epididymitis after their initial operation. We performed a second operation in which the urethra was dilated and the external orifice of the urethra was torn to the coronal sulcus. After that, epididymitis did not recur and satisfactory clinical results were achieved. However, the opening of the coronal sulcus has clear drawbacks. First, there is a significant difference between the appearance of the coronal sulcus and the normal penis, which may affect the psychological health and sexual functions of the patients. Second, urination is sprinkled during the passage, which affects the urination process. However, we believe that the advantage of the coronal sulcus opening is greater than the disadvantage of infertility caused by repeated painful attacks of epididymitis and eventual surgical removal of the PUC and blockage of the vas deferens. Of course, there is also a need to clarify why hypospadias patients with PUC are more likely to have epididymitis than ordinary hypospadias patients. We think that there may be the following reasons: first, patients with PUC may also have dysplasia of ejaculatory orifice, and the anti-reflux mechanism is defective; second, the cystic structure is prone to residual more urine, so it is more prone to infection. These theories need further experimental research. Our study confirmed that reducing reflux can reduce the incidence of epididymitis in these patients, which is to deal with only one pathogenic factor to solve this problem. As for improving the anti-reflux effect of the ejaculatory duct or using other mechanisms to fundamentally solve this kind of disease, it is worth further exploring.

## Conclusion

The objective of eliminating epididymitis was achieved by locating the urethral orifices in the coronary sulcus in three cases of hypospadias with PUC during urethroplasty and dilatating the urethral orifices to the coronary sulcus in four cases of hypospadias with PUC after repeated epididymitis after urethroplasty. So the patients with hypospadias complicated by the presence of a PUC, especially those with a PUC connects to the vas deferens, we advocate opening the urethral orifice near the coronal sulcus rather than through the glans cavernosum to place it in front of the glans, so that the occurrence of epididymitis after urethroplasty may be reduced.

## Data Availability

The datasets used and/or analyzed during the current study are available from the corresponding author on reasonable request.

## Abbreviations

PUC: Prostatic utricle cyst
MAGPI: Meatal advancement and glanuloplasty incorporated
MRI: Magnetic resonance imaging
VCUG: Cystourethrography
MIF: Mullerian tube inhibitor
